# Immunoinformatics-Based Proteome Mining to Develop a Next-Generation Vaccine Design against *Borrelia burgdorferi*: The Cause of Lyme Borreliosis

**DOI:** 10.3390/vaccines10081239

**Published:** 2022-08-02

**Authors:** Kashaf Khalid, Omar Ahsan, Tanwir Khaliq, Khalid Muhammad, Yasir Waheed

**Affiliations:** 1 Clinical & Biomedical Research Center (CBRC), Foundation University Islamabad, Islamabad 44000, Pakistan; kashaf.khalid1997@gmail.com; 2 Department of Medicine, Foundation University Islamabad, Islamabad 44000, Pakistan; omar.ahsan@fui.edu.pk; 3 Department of Molecular Biology, Shaheed Zulfiqar Ali Bhutto Medical University (SZABMU), Islamabad 44000, Pakistan; vc@szabmu.edu.pk; 4 Department of Biology, College of Sciences, United Arab Emirates University, Al Ain 15551, United Arab Emirates; 5 Office of Research, Innovation & Commercialization, Shaheed Zulfiqar Ali Bhutto Medical University (SZABMU), Islamabad 44000, Pakistan

**Keywords:** *Borrelia burgdorferi*, Lyme borreliosis, molecular dynamics simulations, molecular docking, next-generation vaccine

## Abstract

The tick-borne bacterium, *Borrelia burgdorferi* has been implicated in Lyme disease—a deadly infection, formerly confined to North America, but currently widespread across Europe and Asia. Despite the severity of this disease, there is still no human Lyme disease vaccine available. A reliable immunoinformatic approach is urgently needed for designing a therapeutic vaccine against this Gram-negative pathogen. Through this research, we explored the immunodominant proteins of *B. burgdorferi* and developed a novel and reliable vaccine design with great immunological predictability as well as low contamination and autoimmunity risks. Our initial analysis involved proteome-wide analysis to filter out proteins on the basis of their redundancy, homology to humans, virulence, immunogenicity, and size. Following the selection of proteins, immunoinformatic tools were employed to identify MHC class I & II epitopes and B-cell epitopes, which were subsequently subjected to a rigorous screening procedure. In the final formulation, ten common MHC-I and II epitopes were used together with a suitable adjuvant. We predicted that the final chimeric multi-epitope vaccine could invoke B-cell responses and IFN-gamma-mediated immunity as well as being stable and non-allergenic. The dynamics simulations predicted the stable folding of the designed molecule, after which the molecular docking predicted the stability of the interaction between the potential antigenic epitopes and human immune receptors. Our studies have shown that the designed next-generation vaccine stimulates desirable immune responses, thus potentially providing a viable way to prevent Lyme disease. Nevertheless, further experimental studies in a wet lab are needed in order to validate the results.

## 1. Introduction

Lyme disease (or Lyme borreliosis), is triggered by *Borrelia burgdorferi*, a type of bacteria most commonly found in the US and Europe, but spreading to several Asian countries as well [1,2]. By infecting humans through tick bites, the bacterium is rapidly spreading. A study conducted by the CDC says that ~0.5 million people in the US contract Lyme borreliosis every year [3]. Generally, four of the twenty genospecies of *B. burgdorferi* are involved in causing Lyme disease among humans: *B. burgdorferi*, *B. afzelii*, *B. garinii*, and the newly discovered *B. mayonii* [4,5]. In clinical practice, Lyme disease manifests several symptoms, ranging from mild to severe, such as fatigue, cognitive difficulties, muscle pain, and musculoskeletal disorders—sometimes persisting for years [6]. Additionally, Lyme carditis, Arthritis, and Neuroborreliosis have been observed to be among the potentially lethal consequences of untreated *Lyme borreliosis* (LB) [7,8].

An OspA-based vaccine was originally used to treat Lyme disease in 1998 and was successful in creating a humoral response and in blocking transmission, however, it was withdrawn in 2002 due to safety concerns and the requirement for booster doses to maintain immunity [9,10]. Twenty years after that, there are still no human vaccines or FDA-approved pharmaceutical therapies to thwart the infections caused by *B. burgdorferi* [6]. Despite the widespread awareness of Lyme disease, no significant efforts have been made to predict a successful vaccine design [11,12]. However, innovative immunogenic vaccine candidates have been developed for various vector-borne diseases in the past few years [13,14]. A recent study conducted by Doolan et al. has referred to Lyme disease as a “ticking time bomb” [15]. It is therefore imperative to develop effective therapeutic/prophylactic treatments to counteract the deadly menace.

Recent advances in computational immunology and vaccine informatics have made it possible to engineer non-allergenic, non-toxic, and potent multiepitope vaccines against viral, bacterial and infectious diseases mediated by pathogens [16,17]. As the incidence of LB is rapidly climbing and the long-term health consequences of infection are serious [18], computational studies are necessary to design a theoretical vaccine that could be verified for its ability to provide protection, against Lyme disease. The present study is therefore designed to identify the antigenic proteins that can be used to build a comprehensive construct to combat Borrelia-associated diseases by investigating the epitopes of *B. burgdorferi*. Bioinformatics tools were harnessed to project and assess the antigenic, cross-protective, non-allergenic, and non-toxic epitopes in the numerous proteins of *B. burgdorferi*, which were then adjoined with TLR-2 activating adjuvants to enhance lifelong immunity. Through molecular dynamic simulations, the construct’s structural integrity and dynamics were assessed. Furthermore, docking of the construct with the human receptors TLR-1 and TLR-2 was conducted to decipher the binding capacity of the designed construct. We decided to dock TLR 1 and 2 since they are known to play an important role in the pathogenesis of Lyme borreliosis by activating innate immune responses and recognizing proteins from *B. burgdorferi* organisms [19,20]. Lastly, the designed complex was sent for in-silico cloning, followed by immune simulations to evaluate the level of response generated when given as primary, secondary, and tertiary doses of the immune complex. As a result, the current study provides a unique opportunity for researchers to design an efficient vaccine for the treatment of LB.

## 2. Materials and Methods

**Figure 1:**
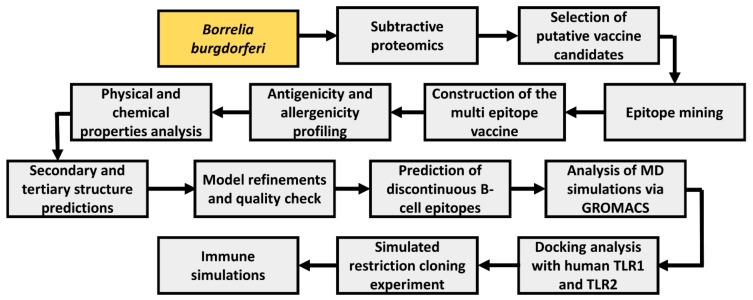
Workflow blueprint employed throughout the study. Study bacteria is depicted in yellow boxes, while study procedures are depicted in gray boxes.

A wide range of computational tools was employed to develop a multiepitope vaccine (MEV) using multiple Borrelia proteins. A general schema adopted in this study is illustrated in Figure 1.

### 2.1. Subtractive Proteomics

We used Uniport to fetch the complete proteome of *B. burgdorferi* (Strain: ATCC 35210) using the Uniport ID UP000001807 [21]. A total of 1290 proteins were downloaded. By utilizing the approach of subtractive proteomics, we shortlisted the target proteins and designed a vaccine based on epitopes against *B. burgdorferi*. Initially, a threshold level of 0.9 was applied to the CD-HIT suite to filter out repetitive or similar proteins [22]. The PSORTb 3.0 tool (Brinkman Laboratory, Simon Fraser University, British Columbia, Canada), which has been identified as the most precise localization predictor tool [23], allowed us to determine the subcellular localization of proteins, excluding those located within the cytoplasm since proteins found within the cytoplasm are not identified by the host immune system; therefore, proteins located outside or within periplasmic membranes are critical for pathogen attachment, invasion, and survival [23]. The residual proteins were sent for consequent downstream analyses.

It is anticipated that virulent proteins might make good vaccine candidates due to their pathogenicity, interactions with host pathways, and conservation through many strains. Accordingly, proteins similar to those deposited in VirulentPred were chosen from proteins selected in previous processes [24]. VirulentPred is a reliable SVM technique that is used to identify proteins showing virulence with a precision of 81.8% [24].

The potential immunogenicity of shortlisted virulent proteins that were not similar to humans and found externally in cytoplasm was determined using VaxiJen (The Jenner Institute Laboratories, Oxford, UK) [25]. Our epitope search only took into account proteins with an antigenicity >0.4 and a molecular mass >110 kDa [26].

### 2.2. Epitope Mining

By using NetCTL’s default threshold of 0.75 for identifying epitopes, we identified proteins that can stimulate cell-mediated immunity [27]. NetCTL uses a method that predicts CTL epitopes capable of interacting with Class I MHC proteins that trigger CTL activation [27]. In order to prevent further infection, the proposed CTL epitopes would induce cellular immunity, inhibiting the growth of pathogen and resulting in the stimulation of memory T cells.

The B-cell receptors and the immunoglobulin generated by triggered B cells detect peptides in antigens. It is important to stimulate the humoral immune response to help clear pathogens through antibody-mediated immunity [28].

For the immune system to become fully activated, helper T lymphocytes (HTL) must also be stimulated. The HTLs recognize epitopes that are loaded onto MHC II proteins on B cells and other APCs. Employing a web tool provided by the IEDB, epitopes were predicted for HTL [29]. This tool compares the results of three methods (SMMalign, Sturniolo, and Combinatorial library) with five million 15-mer peptides found in the UniProt in order to rank predicted epitopes according to their percentile scores [30].

### 2.3. Prediction of IFN-γ Epitopes

Using the IFNepitope web server [31], we predicted the MHC-II epitopes which prompt the interferon-gamma with a precision of 82%. Using machine learning, the server predicts epitope interferon-producing properties and assigns the score of each input epitope based on the support vector machine-based model and motif-based model.

### 2.4. Epitope Screening

Our shortlist of predicted epitopes (CTL and HTL) was further refined through multiple filters, including antigenicity, solubility, and toxicity. For each epitope, we used the online Web servers VaxiJen [25], ToxinPred [32] and peptide solubility calculator [33]. For vaccine design, it was decided to select epitopes predicted to be water-soluble, non-toxic, non-antigenic, and positive binders.

A BLAST search of top-ranking T-cell and B-cell sequences for cross-protection and self-antibody examination was conducted with Uniport-BLAST [34]. Uniport-BLAST searches performed against the uniportkb_human databases revealed protein epitopes that matched predicted self-antigens in human proteins.

### 2.5. Epitope Assemblage

AAY and GPGPG were utilized as linkers to connect the shortlisted epitopes [35]. Using AAY linkers, CTL epitopes were adjoined, whereas HTL epitopes were connected via the GPGPG linkers. Epitopes are separated through these linkers to circumvent junctional epitopes and to enhance the epitope display [36]. Moreover, since TLR-2 is mainly induced by the Borrelia infection, a TLR-2 agonist: Pam3CSK4 (PDB ID: 2Z7X_3_C) was included as an adjuvant to the MEV to increase the immune instigator properties [26]. A linker-EAAK was employed to add adjuvant to the designed construct at its N-terminus.

### 2.6. Conformational B Cell Epitope Analysis

The arrangement of distant residues adjacent to each other as a result of protein folding produces B cell epitopes with discontinuous structures. Approximately, ninety percent of epitopes on B-cells are discontinuous [37]. A refined, validated 3D structure with multiple epitopes was uploaded to the ElliPro server to investigate the existence of the conformational B cell epitopes [37].

### 2.7. Antigenicity and Safety Profiling of the MEV

The antigenic potential was determined using the VaxiJen 2.0 (The Jenner Institute Laboratories, Oxford, UK) [25]. Based on a non-alignment-based algorithm, the server gives the score of antigenicity for the query sequence with 70–90% accuracy AllerTop and AlgPred were employed to forecast the non-allergenic nature of the vaccine [38,39]. We performed a BLAST search [40] on the designed chimera to ensure that its sequence shares no match with the human proteome. Our vaccine construct was also submitted to PBIT server [41] to ensure there was no similarity to commonly found gut microbiota, since microbiota play a very important role in an organism’s homeostasis.

### 2.8. Physicochemical Profiling, Structure Projections, Model Refinement, and Quality-Check

ProtParam(SIB Swiss Institute of Bioinformatics, Geneva, Switzerland.) [42] was harnessed to examine the physicochemical aspects of the MEV, such as the stability index, molecular weight, PI, amino acid conformation, half-life evaluation, and GRAVY index. For the successful presentation of antigenic peptides on MHC, it is essential to know the quality of the secondary and tertiary structure of the vaccine construct during vaccine development [43]. PSIPRED server [44] was utilized for secondary structure extrapolation of MEV sequence. I-TASSER server [45] helped in generating the 3D vaccine construct by using comparative modeling. The C-score ranges between 2 and 5, with a greater score signifying a better model [46].To validate the refined prediction model, Galaxyrefine [47], Molprobity [48], ERRAT [49] and ProSA-web [50] servers were employed.

### 2.9. MD Analysis

Molecular dynamics (MD) analysis is performed to inspect the steadiness of a protein [51]. GROMACS 5.0.5 (the Royal Institute of Technology and Uppsala University, Stockholm Sweden) [52] was used to study the correct folding ability and structural properties of the designed construct. Using OPLS/AA force field, MD analysis was conducted. Next, the steric clashes and geometry of the system were verified using an energy minimization algorithm before simulation. During the equilibration phase, a temperature of 300 K and a pressure of 1 bar were achieved (100 ps). As a final step, the trajectory data produced from the simulation (20 ns) was investigated in terms of RMSD, RMSF, and RMSF of the backbone atom relative deviations generated from the simulation.

### 2.10. Molecular Docking with TLR-1 and TLR-2

Human TLR-1 and 2 are well-understood to play an integral part in Lyme borreliosis and, because of their involvement in triggering innate immune responses by actively recognizing proteins from the *B. burgdorferi* organisms, we selected them for docking studies [19,20]. A docking study analyzes the dynamics of binding between ligands and receptors by computing their affinities. The 3D models of TLR-1 and TLR-2 were saved from the PDB database [53] using the ID 6NIH for TLR-1 and 3A7B for TLR-2.

### 2.11. In-Silico Cloning Experiment

Finally, JCat server [54] was utilized to construct a plasmid harboring the designed vaccine. An organism is selected for providing codon-optimized DNA sequences of interest [54]. We hereby selected *E. coli* (strain K12). Based on its rapid growth, capacity to yield large volumes of protein, and ease of inserting DNA molecules into cells, *E. coli* is deemed a suitable host for cloning [55]. Furthermore, two more parameters were included in the output; the CAI and the percentage of GCs. For optimal performance, CAI should be 1.0 and GC should range between 30 and 70%. We used SnapGene software (Dotmatics, Bishop’s Storford, UK) to conduct the cloning into pET28a (+) expression vector [56]. The multi-epitope sequence was flanked by NdeI and XhoI restriction sites to ensure proper insertion into the plasmid.

### 2.12. Immune Simulations

It is essential that a potent vaccine stimulates an immune response to elicit the same type of long-lasting adaptive immunity induced by an antigen [43]. Therefore, further characterization of the immunogenic potential of the MEV was performed using the C-ImmSim server [57]. Based on PSSM, this agent-based model predicts immune epitopes using machine learning techniques. Three distinct anatomical regions are simulated in this model: (i) the bone marrow, which simulates hematopoietic stem cells, (ii) the thymus, which selects naive T cells devoid of autoprotection, and (iii) a tertiary lymphatic organ, a lymph node. With time steps of 1, 42, and 84, default values were set for all simulation parameters. As a result, four weeks apart, three injections were administered [58].

## 3. Results

### 3.1. Data Assemblage and Proteome Subtraction

Using the Uniprot database, the complete proteome of *B. burgdorferi* (strain: ATCC 35210) comprising 1290 proteins was extracted [21]. Following this, the proteome as a whole was put through a subtractive pipeline. To remove those pathogens that shared the set cut-off sequence, the complete protein sequences were first compared to each other. With a 90% identity threshold, CD-HIT was employed to identify 1131 non-paralogous and non-redundant proteins [22]. In the process of developing vaccines and drugs, these unique sequences serve as excellent starting materials. In order to predict the subcellular localization of non-paralogous proteins, the PSORTb server [23] was utilized. In the server, proteins were categorized based on their locations within the cell, as follows: 100 cytoplasmic membranes, 26 outer-membranes, 12 periplasmic, 8 extracellular, and 893 cytoplasmic proteins (Supplementary File). Since cytoplasmic proteins cannot be accessed by the host immune system and thus might not serve as vaccines, they were eliminated [59]. Conversely, the host immune system can readily identify secretome- or surface-exposed proteins, leading to an efficient immune response [60]. Next, proteins homologous to humans were discarded following which virulent proteins were extracted using VirulentPred web server [24] which uses a reliable SVM technique for the automated identification of virulent proteins. In total, 58 cytoplasmic, 24 outer-membrane, 7 extracellular, and 10 periplasmic proteins were classified as virulent (Supplementary Table S1).

### 3.2. Immunogenicity and Protein Size Analysis

VaxiJen server [25] was utilized to envisage the antigenic potential of the proteins. Resultantly, 22 cytoplasmic membranes, 12 outer membranes, 6 periplasmic proteins, and 2 extracellular proteins were found to be immunogenic. Proteins with antigenic potential were chosen and further subjected to AllerTop [38], AlgPred [39], and Protparam [42] to find out the allergenicity and weight of the shortlisted proteins. We selected those with non-allergenic properties and weights lower than 110 kDa. 110 kDa-sized proteins have been suggested as effective targets [61]. There were six outer membrane proteins that were antigenic, non-allergic, non-similar to human, and weighed <110 kDa. Table 1 shows the selected proteins.

### 3.3. Epitope Mining

The selected proteins contained 60 potential CTL epitopes that were all non-allergenic, non-toxic as well as immunogenic. Using NetCTL 1.2 (DTU Health Tech, Lyngby, Denmark) a combinatorial score for each of the epitopes selected was generated [27]. Using each outer membrane protein, we selected the four most favorable CTL epitopes to construct an MEV. Table 2 represents the short-listed CTL epitopes. 

In contrast, as many as 592 HTL epitopes within the specified range of proteins were determined to be antigenic, non-allergenic, and non-toxic. We evaluated the cytokine-inducing properties, the ability to stimulate IFN production, and their solubility, conservation, and cross-protective properties to shortlist the three most favorable epitopes from the ABC transporter permease, MurJ protein, and YggT family protein for the final assembly of an MEV. A list of the shortlisted HTL epitopes is shown in Table 3.

The selected proteins also yielded 61 potential epitopes from LBL analysis, which were then screened against antigenicity, toxicity, conservancy, and solubility. For the formulation of a multiepitope vaccine, three favorable LBL epitopes from each of the TsaE, FliP, and MreD proteins were chosen based on their safety profile, antigenicity, probability scores, and non-allergenic nature. Table 4 shows the LBL epitopes that have been shortlisted for vaccine construction.

### 3.4. Designing of the MEV

**Figure 2:**
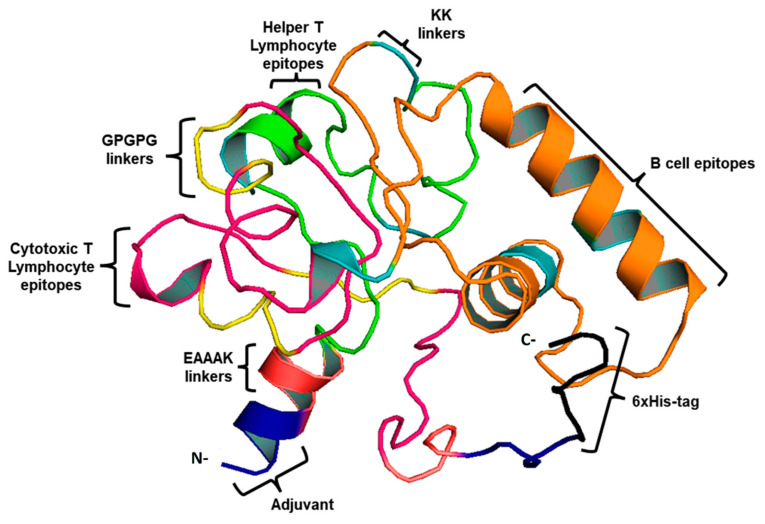
3D vaccine chimera. The vaccine adjuvant is represented by the blue part, the EAAAK linker by red, CTL epitopes by pink, and AAY and GPGPG linkers are indicated by yellow. Whereas, HTL epitopes are indicated by the green color and LBL by the orange. Black represents the 6-His tag.

This study used four CTL, three HTL epitopes, and three LBL epitopes to develop a vaccine. An AAY and GPGPG linker were used to fuse MHC-I and II epitopes. During infection with *B. burgdorferi*, TLR-2 expression is increased, therefore, EAAK linkers were used to merge Pam3CSK4 chain C to the N-terminus of the vaccine construct to increase TLR-2 expression. [26]. As a result of this, a vaccine construct consisting of 419 residues that includes epitopes for both B-cells and T-cells was created (Figure 2).

### 3.5. Antigenicity, Allergenicity, and Safety Profiling of the MEV

We used the VaxiJen [25] and AllerTop [38] servers to decipher the antigenic and allergenic profiles of the MEV, respectively. VaxiJen assessed an antigenic score of 1.114 for the vaccine constructs attached with adjuvants, and the non-adjuvanted vaccine construct showed a score of 1.08. These data serve as a measure of the potential of designed chimera to trigger a protective immune response. AllerTop showed no sign of similarities with allergens for either of the construct. In addition, the *Homo sapiens* and the gut microbiota proteins exhibited no similarity to the multi-epitope vaccine.

### 3.6. Physicochemical Analysis

An adjuvanted vaccine was computed physicochemically using the ProtParam tool (SIB Swiss Institute of Bioinformatics, Geneva, Switzerland) [42]. The construct was characterized by a molecular weight of 18.4 kDa and a theoretical PI of 10.0; additionally, its instability index was 17.10. The half-life of the construct was determined to be more than 20 h and 10 h for yeast and *E. coli* respectively. The thermostability is calculated based on the aliphatic index, which was calculated to be 70.09, whereas a GRAVY value was calculated to be −1.345.

### 3.7. Projection of Secondary Structure

**Figure 3:**
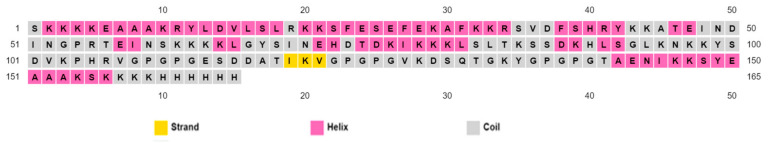
Secondary folding of the proposed chimera as projected by the PSIPRED webserver.

PSIPRED was used to generate the secondary structure [44]. There were 45.1% alpha helices, 42.1% coils, and 12.8% beta strands in the vaccine structure (Figure 3).

### 3.8. Projection, Refinement, and Evaluation of 3D Structure

A chimeric protein tertiary structure model was constructed by the I-TASSER server. Based on their Z-scores (ranging from 1.28 to 3.58), all 10 selected templates indicated better alignment. There were five predicted models with C-scores ranging from 4.15 to 1.15. Typically, C-scores range between −5 and 2, where higher values indicate greater confidence in the prediction. Further refinement was carried out on the model with the highest C-score from homology modeling. TM-score was reported as 0.55 ± 0.15 with RMSD estimated as 7.7 ± 4.3 Å. According to the TM-score, two structures are structurally similar if their scores are similar [62]. TM-scores > 0.5 represent topologies of correct structure, and TM-scores < 0.17 indicate random similarity. Moreover, protein length is not a factor in determining these cut-off values.

**Figure 4:**
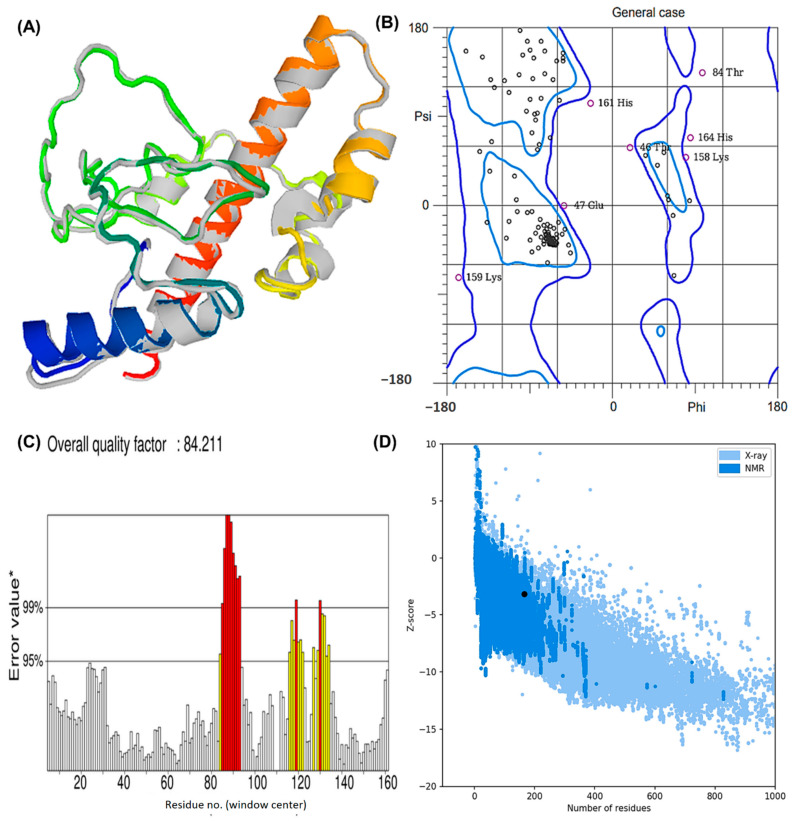
The modeled assembly and the quality check investigation of the designed MEV against Lyme borreliosis (**A**) refined model after GalaxyRefine and ModRefiner. The gray parts of the structure represent the areas refined by the tools (**B**) Rama plot analysis exhibiting residues in the favored, allowed, and disallowed region. Areas encircled by the light blue and dark blue colors are the favoured and allowed regions respectively. (**C**) ERRAT score depicting a quality factor of 84.21 (**D**) Z-plot showing a Z-score of −3.18.

The structure obtained by I-TASSER was refined using ModRefiner (University of Michigan, Ann Arbor, Michigan, United States) and then sent to GalaxyRefine (ELIXIR Node, Copenhagen, Denmank) for further refinement. The “crude” vaccine model was refined using ModRefiner, and five models were further optimized using GalaxyRefine. The GDT-HA quality score for model 5 was the highest among all refined models, along with RMSD (0.512), MolProbity (2.094), and RMSD (0.572) (Figure 4A). Scores for conflict were 14.2, poor rotamers was 0.70, and the Ramachandran plot was 93.3%. A vaccine model based on this model was chosen for further analysis.

Based on Ramachandran plot analysis, the refined tertiary structures of vaccine constructs were validated using RAMPAGE. In the results, the favored region represented 92.25% of the residues, the allowed region represented 5.63%, and the disallowed region represented 2.11% (Figure 4B). There were 92.25% residues found in the favored region, 5.63% in the allowed region, and 2.11% in the disallowed region. The refined model uploaded to ERRAT revealed an overall quality factor of 84.21% (Figure 4C), whereas ProSA-web generated a Z-score of −3.18. According to the results, the Z-score obtained corresponded to a wide array of commonly found proteins of equivalent size, therefore satisfying the validation goal (Figure 4D).

### 3.9. Discontinuous BCEs

An analysis of the 3D model of the MEV was performed to project discontinuous B-cell epitopes. Ellipro (NIAID, Maryland, USA) predicted that 77 residues were located in four discontinuous B-cell epitopes of adjuvanted vaccines, with scores ranging from 0.61 to 0.84. The size of conformational epitopes ranged between fifteen and 33 residues. (Supplementary Table S2)

### 3.10. Analysis of MD Simulations Using GROMACS

We ran MD simulations using the GROMACS, a Linux-based software to determine whether the vaccine construct is stable. The vaccine construct weighed 47,036.917 amu after the application of an OPLS-AA force field. GROMACS was used to add 28,569 water molecules to the system by making use of the integrated Spectro tool. A charge of 3.000e was calculated on the protein. The neutralization was achieved by replacing three water molecules with three CL ions at atoms 39763, 72889, and 20449.

**Figure 5:**
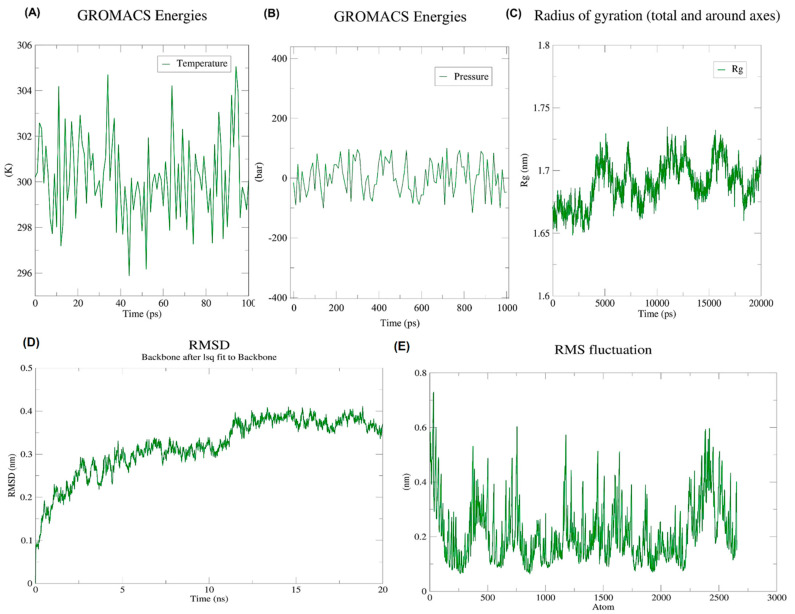
Illustration of the progress of dynamic simulations (**A**) Temperature fluctuations throughout simulations. Within 100 ps, the system temperature reached 300 K with minimal fluctuations; (**B**) Pressure fluctuations throughout simulations. On the pressure graph, the average pressure is indicated as −1.01321 bar for 100 ps; (**C**) RoG. An investigation of the radius of gyration of the vaccine construct shows that it has the potential to maintain its compact form during simulations; (**D**) Backbone RMSD plot. According to the RMSD graph, the RMSD goes up to ~0.4 nm and is mostly sustained afterward, thus indicating the minimum structural deviation of the vaccine construct during the 20 ns simulation; (**E**) RMSF graph. Side chains plotted by RMSF show areas with high flexibility in the form of peaks.

During an energy minimization exercise of 50,000 steps, the steepest descents converged to form a force of <1000 kJ/Mol after 1576 steps. During energy minimization, the average potential energy of the system was determined to be −1.57303 × 10^6^ kJ/mol with a total drift of −157,771 kJ/mol. The potential energy of the system was derived to be −11.6187445× 10^6^ kJ/mol. A 50,000 step NPT experiment revealed that the average temperature was 299.73 K with a total drift of 1.2953 K (Figure 5A), the average pressure was 0.97030862 bar with a total drift of −7.48762 bar (Figure 5B), and the average density was 1018.94 kg/m^3^, with a drift of 1.93 kg/m^3^.

After 20 ns of simulations, trajectory analysis was performed. The protein structure remained stable during MD simulations which was evident by its radius of gyration which did not fluctuate much over the course of time (Figure 5C). According to RMSD backbone analysis, the RMSD values increased by up to 0.6 nm and remained constant during the simulation period (Figure 5D). Meanwhile, RMSFs describe regions that exhibit high flexibility (Figure 5E).

### 3.11. Molecular Docking Analysis

**Figure 6:**
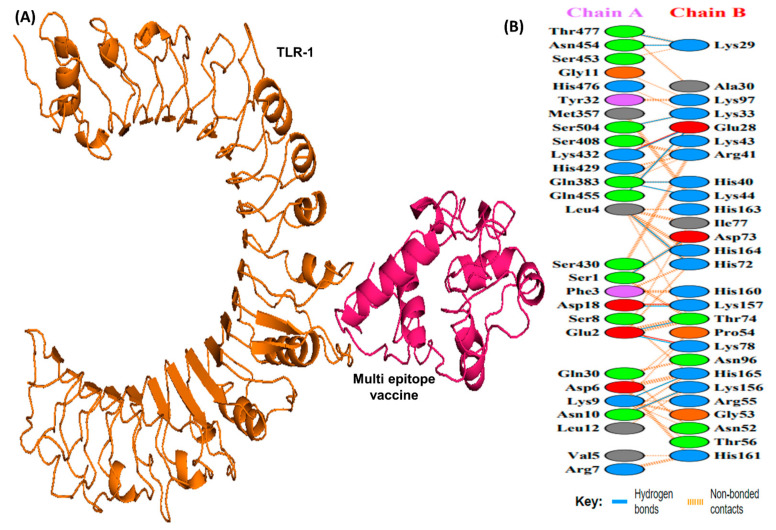
A schematic depicting the docked complex of TLR-1 and vaccine and residue interactions. (**A**) The TLR-1 receptor is shown in orange and the vaccine (in hot pink) is attached to it. (**B**) Interface residue interaction. There are 14 hydrogen bonds depicted in blue lines. Residues are shown in multiple colors to show their properties e.g positive (blue); negative (red); neutral (orange); aliphatic (gray); aromatic (purple).

**Figure 7:**
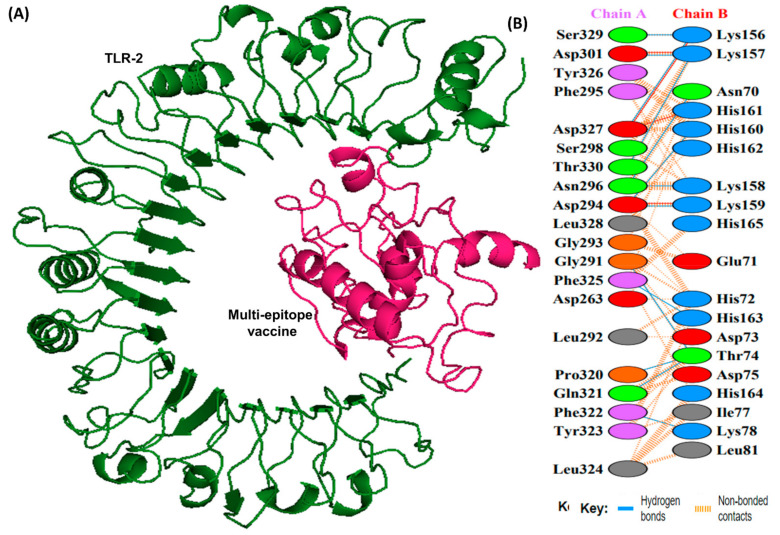
A schematic depicting the docked complex of TLR-2 with vaccine and their residue interactions (**A**) The TLR-2 is illustrated in forest green color, and the vaccine (in hot pink) is displayed bonded to it (**B**) Interface residue interaction. There are 13 hydrogen bonds between the docked complex depicted in blue lines. Residues are shown in multiple colors to show their properties e.g., positive (blue); negative (red); neutral (orange); aliphatic (gray); aromatic (purple).

Docking studies were conducted between the multi-epitope vaccine construct and the human Toll-like receptor. Since TLR-1 and 2 are particularly sensitive to bacterial components on extracellular surfaces [19], they were chosen for docking. Docking with HADDOCK is based on biochemical and biophysical interaction data [63]. For TLR-1, HADDOCK clustered 383 structures in 11 clusters, representing 95.75% of the water-refined models HADDOCK generated, whereas for TLR-2, HADDOCK clustered 393 structures in 3 clusters, representing 98.25% of the water-refined models HADDOCK generated (Figure 6 and Figure 7). The results of the docking are presented in Table 5.

The HADDOCK score depicts a substantial affinity between the receptor and the vaccine, whereas a negative score indicates better docking. There were 27 hydrogen bonds, 7 salt bridges, and 353 non-bonded contacts analyzed (Table 5). Binding affinity or the Gibbs free energy (ΔG) of a complex is crucial to determining whether or not certain conditions in a cell will allow interaction to occur. We analyzed the binding affinity of the docked complex using PRODIGY web servers. The G-values of the vaccine construct were calculated to be −12.1 for TLR-1 and −12.0 for TLR-2. Furthermore, the dissociation constant (kD) among vaccine-TLR1 and vaccine-TLR2 complexes was 1.3 × 10^−9^ and 1.5 × 10^−9^, respectively. Due to the negative ΔG, our results show that the docked complex is energetically viable.

### 3.12. Restriction Cloning In-Silico Experiment 

A DNA sequence containing optimized parameters was generated using the JCAT server. With a CAI of 1.0, increased expression was highly likely. GC content was found to be 42.42%, falling within the ideal values (30–70%). An expression construct pET-28a (+) containing the MEV insert was created using SnapGene (GSL Biotech LLC, Chicago, IL, USA) [56].

### 3.13. Immune Simulations Experiment

**Figure 8:**
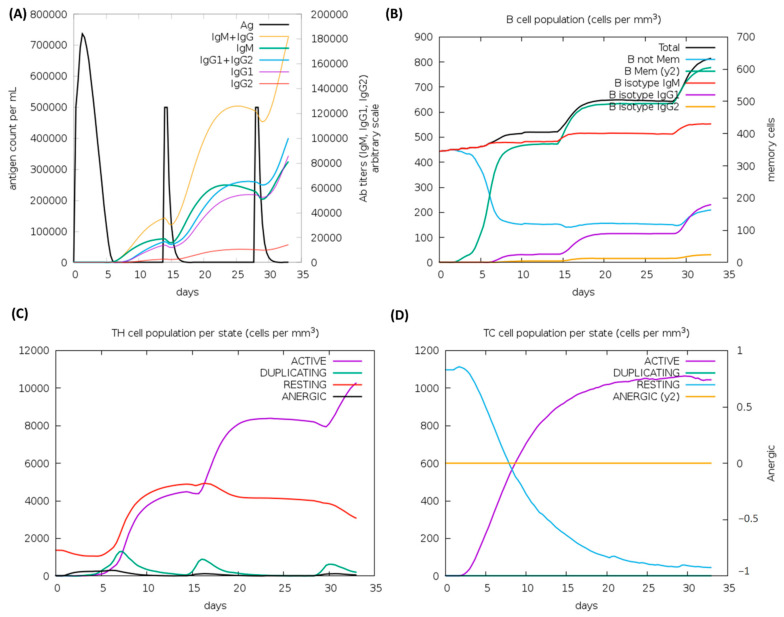
C-ImmSim analysis of a chimeric peptide immune simulation (**A**) Antibody production after antigen injections (black vertical lines); subclass immunoglobulins are displayed in the form of colored peaks (**B**) The alterations to B-cell populations following three injections. (**C**) Changing populations of T-helper cells, and (**D**) the number of T-cytotoxic cells per state following an injection. T-cells in the resting state are not exposed to antigens, while those in the anergic state are tolerant to antigens due to repeated exposures.

According to the outcomes of the immune system simulations generated by the C-ImmSim, a noticeable increment in secondary immune responses was similar to the actual immune responses. The initial response was characterized by a marked level of IgM. It is observed that B-cell counts, IgG1 + IgG2 antibodies, and IgG + IgM antibodies increased in secondary and tertiary immune responses, while levels of antigen reduced (Figure 8A,B). These findings suggest the improvement of immunity, memory, and subsequently, a stronger clearance of the antigen upon re-exposure (Figure 8C). The same extraordinary immune response was also found in the helper and cytotoxic cell populations (Figure 8C,D). Following repeated injections (given four weeks apart), IgG1 levels increased while IgM levels declined, with IFN-γ levels and TH cell levels remaining elevated during the exposure. In this analysis, the intrinsic antigenic properties of the designed construct are thus confirmed.

## 4. Discussion

As reported by the CDC, Lyme borreliosis cases have tripled in the US, becoming the most prevalent vector-transmitted disease, causing about 476,000 cases per year [3,64]. World Health Organization states that Lyme borreliosis is the most rampant and highly infectious vector-associated disease across Europe [65]. With rapid spread and cases reported across Australia, Europe, North America, Asia, and Africa, the threat is rapidly becoming a global health issue with an epidemic potential [66,67]. A recent study conducted by Doolan et al. has referred to Lyme disease as a “ticking time bomb” [15]. In spite of the fact that antibiotic treatments are available for the disease, they are not viable countermeasures on their own not only due to the fact that drug resistance is a risk and that antibiotic treatment results are mainly unsatisfactory but also due to the fact that the *B. burgdorferi* disease is initially hard to detect and only early diagnosis will allow for antibiotic treatment [68,69]. Many studies have called upon scientists to urgently devise effective treatment strategies against Lyme disease [70,71]. In spite of the widespread prevalence, there is no vaccine that has been translated for human use against LB. Subunit vaccines against several bacteria have gained much attention recently since they possess better safety profiles and can be made logistically easier to provide [72]. The use of epitope-based vaccines provides a novel way to produce an immune response with increased safety, as well as a prospect to logically contrive epitopes for increased effectiveness and protection [73].

A combination of immunoinformatics and subtractive proteomics techniques was used in this study to identify proteins against *B. burgdorferi* infection and design multiple epitope-based vaccines against it [74]. We started by analyzing the complete proteome of *B. burgdorferi* from the Uniprot database. To remove pathogens whose sequences share the set cut-off value, the complete protein sequences of the pathogens were first compared to one another. In addition, PSORTb classified the proteins according to their subcellular location. As a next step, virulent proteins were determined using the VirulentPred webserver. Antigenicity of the short-listed protein was predicted by the Vaxijen server, following which the small-sized proteins were filtered to obtain proteins that are not only antigenic in nature but also smaller in size (>110 kDa). The proteins thus narrowed down were subsequently used for epitope mapping. Using several web-based servers and databases, the epitopes of CTL, HTL, and B-cells within vaccine candidate proteins were predicted. In order for a vaccine design to be efficient, it should contain epitopes from CTLs, HTLs, and B-cells [17]. Since B-cells are important for antibody production, we included B-cell epitopes. A humoral response can be overcome by memory B-cells through the production of antigens, whereas immunity mediated by cells (T-cell immunity) can last a lifetime [75]. By releasing specific anti-infective cytokines and identifying and killing pathogen-infected cells, CTLs inhibit the dissemination of pathogens. As a final step, the CTL, HTL, and B-cell epitopes were combined using AAY, GPGPG, and KK linkers, respectively. By using linkers, vaccines can be folded, stabilized, and expressed more effectively [76]. Next, adjuvant coupling is required to boost the immunogenicity, stability, and durability of multiepitope-based vaccines [77]. Adjuvant (TLR-2 agonist: Pam3CSK4) was attached to the 5′ and 3′ prime sites through the EAAAK linkers. By linking the adjuvant with the epitope and the EAAAK linker, the bi-functional fusion protein can be effectively separated [78]. The overall length of the vaccine was found to be 18,473.02, and it appeared to have nil similarity with any protein segments in Homo sapiens. In addition, there was excellent solubility when overexpressed in *E. coli*, making it convenient for the protein to bind to the host. The high stability index indicates that the protein will remain stable after it is expressed, thereby increasing its potential for use. Having a hypothetical pI of 10.0, the proposed model was found to be physiologically alkaline having a stable pH. Also, the aliphatic index and GRAVY score reflected the thermal stability and hydrophilicity, respectively. It was found that half-lives of >30 h in vitro, >20 h in vivo, and >10 h in yeast are in agreement with previously published data. Further, the chimera developed proved to be flexible, nonallergenic, nontoxic, highly antigenic, and immunogenic. Based on these results, the proposed vaccine design was capable of eliciting an adequate immune response free from side effects. A comprehensive understanding of the tertiary structure of proteins can be gained by predicting the spatial features of the main protein components, which then allows for studying the functions of proteins, the interactions among ligands and other components, and the dynamics of proteins. Therefore, modeling the vaccine’s 3D structure enabled us to analyze its desirable properties. There were several computational methods employed, generally, the estimated structure appeared to be reasonable.

For a candidate vaccine to be conveyed efficiently in the host, the immune receptors (TLR-1 and 2) and the proposed ensemble need to be connected in a steady manner. In addition to showing that the vaccine could remain folded in a very stable manner under lifelike conditions, the molecular docking analysis also confirmed that the chimera and immune receptor exhibit strong interactions. In MEV-TLR complexes, a large number of hydrogen bonds were detected during the protein-protein docking. Considering these findings, it seems likely that the proposed MEV has the ability to bind effectively to immune receptors.

After being bound to human immune receptors and delivered into the body, a vaccine construct was assessed for its ability to simulate the host immune system. The vaccine should theoretically provoke both arms of the immune system namely, cell and antibody-mediated immune reactions. During the immune simulation validation of the designed MEV, IFN--γ was produced at the highest level. Ultimately. Optimization of codons was achieved to optimize protein expression in the *E. coli* system. After insertion of Xhol and Ndel sites, the transcript of the design was expressed in *E. coli*. Research involving overexpressed proteins requires that recombinant proteins be solubilized. As predicted by the design model, the overexpressed solubility within the *E. coli* system was quite acceptable.

There have been no successful attempts at developing a potent vaccine against *B. burdogferi* to date except for one that was retracted due to undesirable properties in 1998. To determine the putative vaccine candidates against bacterial infection, a number of clinical trials are underway with the aim of conducting human trials shortly [1]. In contrast to traditional vaccines and other reported vaccines, the MEV we hypothesized in this study, has several advantages: (i) it possesses B-cell, T-cell, and HTL epitopes from antigenic proteins, thus stimulating cellular and humoral immunity; (ii) the vaccine possesses different epitopes meant to target different Histocompatibility Antigens and thus can be used to identify T-cell receptors that are effective in different populations; (iii) a single vaccine may contain multiple proteins that tend to aggregate into one fragment, hence increasing the effectiveness of the vaccine; iv) Auto-immunity is reduced due to the non-human proteins covering the epitope, while the other proteins are exempt; (v) The vaccine can also provide lasting immunity because it is combined with an adjuvant. Since the proposed vaccine is formulated with CTL, HTL, and B-cell epitopes as well as a suitable adjuvant, it may result in an innate-adaptive immunity in the host. The vaccine is therefore an excellent candidate for treating Lyme borreliosis.

## 5. Conclusions

Presently, in-silico techniques for identifying proteins and promiscuous antigens found in pathogens, as well as peptides, are heavily used in the process of vaccine development. Therefore, using immunoinformatic tools, we constructed a prospective vaccine ensemble that encoded for multiple B-cell and T-cell epitopes (CTL and HTL). According to our in-silico assessments, the vaccine initiated stable, strongly antigenic, non-allergenic, and non-toxic humoral and cellular responses. Further evidence of steady interaction between the designed construct and human TLRs was confirmed by the molecular docking analysis. This chimeric vaccine peptide may offer a complementary way to eliminate borreliosis. Due to the cross-protective effect of the engineered vaccine candidate, other borrelia species that cause disease throughout the world might also be tackled. However, further investigational wet lab experiments are necessary to verify the potential utility of the proposed construct as an anti-borreliosis treatment option.

## Figures and Tables

**Table 1:** Proteins shortlisted for multiple epitope prediction using proteome analysis of *B. burgdorgeri*.

Protein Name	Protein ID	Antigenicity (>0.4)	Virulence	Allergenicity	Homology to Humans	Size (kDa)
TsaE	WP_002556784.1	0.68	✓	✗	Not found	15.4
FliP	WP_002556874.1	0.71	✓	✗	Not found	29.0
ABC transporter permease	WP_002557338.1	0.53	✓	✗	Not found	27.6
MreD	WP_002656052.1	0.71	✓	✗	Not found	20.9
YggT family protein	WP_002656831.1	0.63	✓	✗	Not found	22.2
MurJ	WP_002657239.1	0.64	✓	✗	Not found	58

**Table 2:** A shortlist of CTL epitopes to be used in the final vaccine design.

CTL Epitope (9-mer)	Protein ID	Combined Score	VaxiJen	Toxin	Conservancy in *B. burgdorfei* sp.	Solubility
KSEKKMINF	WP_002556784	0.9186	0.5478	No	100%	Good
TTNGLNFPF	WP_002556874	0.8427	1.3018	No	100%	Good
DLGIILLQY	WP_002557338	0.8397	0.8354	No	100%	Good
IIFAKPIMY	WP_002657239	0.8012	0.5076	No	100%	Good

**Table 3:** A shortlist of HTL epitopes to be used in the final vaccine design.

Epitope (9-mer)	Protein ID	Percentile Rank	Antigenicity	Toxin	IFN Epitope	Conservancy	Solubility
IILLQYLGI	WP_002557338	0.01	0.6290	No	Positive	100%	Good
FQWDVGFSF	WP_002657239	0.01	1.82	No	positive	100%	Good
ILILIRILL	WP_002656831	0.01	1.2411	No	positive	100%	Good

**Table 4:** A list of LBL epitopes to be used in the final vaccine design.

Linear B Cell Epitope (15-mer)	Protein	Probability Score	Antigenicity	Toxin	Conservancy (%)	Solubility
IALSIVPKDRLFSLTF	WP_002556784.1	0.85	0.7432	No	100	Good
MGMIMLPPVMISLPFK	WP_002556874.1	0.92	1.2510	No	100	Good
YFTGLPLGFFVFGYTI	WP_002656052.1	0.75	0.9086	No	100	Good

**Table 5:** Docking analysis of TLR-1 and TLR-2 to the vaccine construct.

Docking Analysis	TLR-1	TLR-2
HADDOCK Parameters		
HADDOCK score	−79.2 ± 14.1	−103.0 ± 2.5
Z-Score	−1.3	−0.8
RMSD	1.2 ± 1.5	0.3 ± 0.1
Van der Waals energy	−81.4 ± 2.4	−82.7 ± 4.1
Electrostatic energy	−332.3 ± 50.7	−335.2 ± 49.5
Desolvation energy	−14.0 ± 10.2	−51.2 ± 10.6
Buried Surface Area	2567.8 ± 210.5	1938.2 ± 63.0
Binding affinity and kD prediction		
ΔG (kcal mol^−1^)	−12.1	−12.0
Kd (M) at 25.0 °C	1.3 × 10^−9^	1.5 × 10^−9^
Number of Interfacial Contacts (ICs) per property		
Charged-charged	22	14
Charged-polar	42	15
Charged-apolar	23	36
Polar-polar	3	2
Polar-apolar	9	8
Apolar-apolar	3	5
Protein-protein interface interaction statistics		
Salt bridges	3	4
Hydrogen bonds	14	13
No. of non-bonded contacts	149	204

## Data Availability

Not applicable.

## Supplementary Materials

The following supporting information can be downloaded at: https://www.mdpi.com/article/10.3390/vaccines10081239/s1, Table S1: Shortlisting of the putative vaccine candidates from the borrelia burgdorferi proteome, Table S2. Conformational B cells; Supplementary File S1. Subcellular localization of proteins.

## References

[1] Centers for Disease Control and Prevention (2017). Lyme Disease Vaccine | Lyme Disease | CDC. https://www.cdc.gov/lyme/prev/vaccine.html.

[2] Steere A.C., Strle F., Wormser G.P., Hu L.T., Branda J.A., Hovius J.W.R., Li X., Mead P.S. (2016). Lyme Borreliosis. Nat. Rev. Dis. Prim..

[3] How Many People Get Lyme Disease? | Lyme Disease | CDC. https://www.cdc.gov/lyme/stats/humancases.html.

[4] Baranton G., Assous M., Postic D. (1992). Three Bacterial Species Associated with Lyme Borreliosis. CLinical and Diagnostic Implications. Bull. Acad. Natl. Med..

[5] Kingry L.C., Batra D., Replogle A., Rowe L.A., Pritt B.S., Petersen J.M. (2016). Whole Genome Sequence and Comparative Genomics of the Novel Lyme Borreliosis Causing Pathogen, Borrelia Mayonii. PLoS ONE.

[6] Rebman A.W., Aucott J.N. (2020). Post-Treatment Lyme Disease as a Model for Persistent Symptoms in Lyme Disease. Front. Med..

[7] Kamp H.D., Swanson K.A., Wei R.R., Dhal P.K., Dharanipragada R., Kern A., Sharma B., Sima R., Hajdusek O., Hu L.T. (2020). Design of a Broadly Reactive Lyme Disease Vaccine. NPJ Vaccines.

[8] Schrestha K., Kadkhoda K. (2022). Early Lyme Disease-Associated Guillain Barre Syndrome: A Case Report. IDCases.

[9] Lyme Disease Vaccines | NIH: National Institute of Allergy and Infectious Diseases. https://www.niaid.nih.gov/diseases-conditions/lyme-disease-vaccines.

[10] Wormser G.P. (2022). A Brief History of OspA Vaccines Including Their Impact on Diagnostic Testing for Lyme Disease. Diagn. Microbiol. Infect. Dis..

[11] Kitsou C., Pal U. (2022). Vaccines Against Vector-Borne Diseases. Methods Mol. Biol..

[12] Badawi A., Shering M., Rahman S., Lindsay L.R. (2017). A Systematic Review and Meta-Analysis for the Adverse Effects, Immunogenicity and Efficacy of Lyme Disease Vaccines: Guiding Novel Vaccine Development. Can. J. Public Health.

[13] Fatima I., Ahmad S., Abbasi S.W., Ashfaq U.A., Shahid F., Tahir ul Qamar M., Rehman A., Allemailem K.S. (2022). Designing of a Multi-Epitopes-Based Peptide Vaccine against Rift Valley Fever Virus and Its Validation through Integrated Computational Approaches. Comput. Biol. Med..

[14] Tahir Ul Qamar M., Ismail S., Ahmad S., Mirza M.U., Abbasi S.W., Ashfaq U.A., Chen L.L. (2021). Development of a Novel Multi-Epitope Vaccine Against Crimean-Congo Hemorrhagic Fever Virus: An Integrated Reverse Vaccinology, Vaccine Informatics and Biophysics Approach. Front. Immunol..

[15] Doolan B.J., Christie M., Dolianitis C. (2019). A Ticking Time Bomb: A Case of Lyme Disease. Australas. J. Dermatol..

[16] Bidmos F.A., Siris S., Gladstone C.A., Langford P.R. (2018). Bacterial Vaccine Antigen Discovery in the Reverse Vaccinology 2.0 Era: Progress and Challenges. Front. Immunol..

[17] Zhang L. (2018). Multi-Epitope Vaccines: A Promising Strategy against Tumors and Viral Infections. Cell. Mol. Immunol..

[18] Bobe J.R., Jutras B.L., Horn E.J., Embers M.E., Bailey A., Moritz R.L., Zhang Y., Soloski M.J., Ostfeld R.S., Marconi R.T. (2021). Recent Progress in Lyme Disease and Remaining Challenges. Front. Med..

[19] Cassiani-Ingoni R., Cabral E.S., Lünemann J.D., Garza Z., Magnus T., Gelderblom H., Munson P.J., Marques A., Martin R. (2006). Borrelia Burgdorferi Induces TLR1 and TLR2 in Human Microglia and Peripheral Blood Monocytes but Differentially Regulates HLA-Class II Expression. J. Neuropathol. Exp. Neurol..

[20] Cabral E.S., Gelderblom H., Hornung R.L., Munson P.J., Martin R., Marques A.R. (2006). Borrelia Burgdorferi Lipoprotein-Mediated TLR2 Stimulation Causes the down-Regulation of TLR5 in Human Monocytes. J. Infect. Dis..

[21] UniProt Consortium T. (2018). UniProt: The Universal Protein Knowledgebase. Nucleic Acids Res..

[22] Li W., Godzik A. (2006). Cd-Hit: A Fast Program for Clustering and Comparing Large Sets of Protein or Nucleotide Sequences. Bioinformatics.

[23] Yu N.Y., Wagner J.R., Laird M.R., Melli G., Rey S., Lo R., Dao P., Cenk Sahinalp S., Ester M., Foster L.J. (2010). PSORTb 3.0: Improved Protein Subcellular Localization Prediction with Refined Localization Subcategories and Predictive Capabilities for All Prokaryotes. Bioinformatics.

[24] Garg A., Gupta D. (2008). VirulentPred: A SVM Based Prediction Method for Virulent Proteins in Bacterial Pathogens. BMC Bioinform..

[25] Doytchinova I.A., Flower D.R. (2007). VaxiJen: A Server for Prediction of Protective Antigens, Tumour Antigens and Subunit Vaccines. BMC Bioinform..

[26] Khan S., Ali S.S., Zaheer I., Saleem S., Ziaullah, Zaman N., Iqbal A., Suleman M., Wadood A., Rehman A.U. (2022). Proteome-Wide Mapping and Reverse Vaccinology-Based B and T Cell Multi-Epitope Subunit Vaccine Designing for Immune Response Reinforcement against Porphyromonas Gingivalis. J. Biomol. Struct. Dyn..

[27] Larsen M.V., Lundegaard C., Lamberth K., Buus S., Lund O., Nielsen M. (2007). Large-Scale Validation of Methods for Cytotoxic T-Lymphocyte Epitope Prediction. BMC Bioinform..

[28] Saha S., Raghava G.P.S. (2006). Prediction of Continuous B-Cell Epitopes in an Antigen Using Recurrent Neural Network. Proteins Struct. Funct. Bioinform..

[29] Zhang Q., Wang P., Kim Y., Haste-Andersen P., Beaver J., Bourne P.E., Bui H.-H., Buus S., Frankild S., Greenbaum J. (2008). Immune Epitope Database Analysis Resource (IEDB-AR). Nucleic Acids Res..

[30] Jensen K.K., Andreatta M., Marcatili P., Buus S., Greenbaum J.A., Yan Z., Sette A., Peters B., Nielsen M. (2018). Improved Methods for Predicting Peptide Binding Affinity to MHC Class II Molecules. Immunology.

[31] Validi M., Karkhah A., Prajapati V.K., Nouri H.R. (2018). Immuno-Informatics Based Approaches to Design a Novel Multi Epitope-Based Vaccine for Immune Response Reinforcement against Leptospirosis. Mol. Immunol..

[32] Gupta S., Kapoor P., Chaudhary K., Gautam A., Kumar R., Raghava G.P.S. (2015). Peptide Toxicity Prediction. Methods Mol. Biol..

[33] Peptide Solubility Calculator. https://pepcalc.com/peptide-solubility-calculator.php?msclkid=f30d6654d10711ec8686f820ae9d4dad.

[34] BLAST. https://www.uniprot.org/blast/.

[35] Sanami S., Azadegan-Dehkordi F., Rafieian-Kopaei M., Salehi M., Ghasemi-Dehnoo M., Mahooti M., Alizadeh M., Bagheri N. (2021). Design of a Multi-Epitope Vaccine against Cervical Cancer Using Immunoinformatics Approaches. Sci. Rep..

[36] Khan M., Khan S., Ali A., Akbar H., Sayaf A.M., Khan A., Wei D.Q. (2019). Immunoinformatics Approaches to Explore Helicobacter Pylori Proteome (Virulence Factors) to Design B and T Cell Multi-Epitope Subunit Vaccine. Sci. Rep..

[37] Ponomarenko J., Bui H.H., Li W., Fusseder N., Bourne P.E., Sette A., Peters B. (2008). ElliPro: A New Structure-Based Tool for the Prediction of Antibody Epitopes. BMC Bioinform..

[38] Dimitrov I., Bangov I., Flower D.R., Doytchinova I. (2014). AllerTOP v.2—A Server for In Silico Prediction of Allergens. J. Mol. Model..

[39] Saha S., Raghava G.P.S. (2006). AlgPred: Prediction of Allergenic Proteins and Mapping of IgE Epitopes. Nucleic Acids Res..

[40] McGinnis S., Madden T.L. (2004). BLAST: At the Core of a Powerful and Diverse Set of Sequence Analysis Tools. Nucleic Acids Res..

[41] Shende G., Haldankar H., Barai R.S., Bharmal M.H., Shetty V., Idicula-Thomas S., Hancock J. (2017). PBIT: Pipeline Builder for Identification of Drug Targets for Infectious Diseases. Bioinformatics.

[42] Artimo P., Jonnalagedda M., Arnold K., Baratin D., Csardi G., De Castro E., Duvaud S., Flegel V., Fortier A., Gasteiger E. (2012). ExPASy: SIB Bioinformatics Resource Portal. Nucleic Acids Res..

[43] Omoniyi A.A., Adebisi S.S., Musa S.A., Nzalak J.O., Bauchi Z.M., Bako K.W., Olatomide O.D., Zachariah R., Nyengaard J.R. (2022). In Silico Design and Analyses of a Multi-Epitope Vaccine against Crimean-Congo Hemorrhagic Fever Virus through Reverse Vaccinology and Immunoinformatics Approaches. Sci. Rep..

[44] McGuffin L.J., Bryson K., Jones D.T. (2000). The PSIPRED Protein Structure Prediction Server. Bioinformatics.

[45] Roy A., Kucukural A., Zhang Y. (2010). I-TASSER: A Unified Platform for Automated Protein Structure and Function Prediction. Nat. Protoc..

[46] Zhang Y. (2008). I-TASSER Server for Protein 3D Structure Prediction. BMC Bioinform..

[47] Heo L., Park H., Seok C. (2013). GalaxyRefine: Protein Structure Refinement Driven by Side-Chain Repacking. Nucleic Acids Res..

[48] Chen V.B., Arendall W.B., Headd J.J., Keedy D.A., Immormino R.M., Kapral G.J., Murray L.W., Richardson J.S., Richardson D.C. (2010). MolProbity: All-Atom Structure Validation for Macromolecular Crystallography. Acta Crystallogr. Sect. D Biol. Crystallogr..

[49] Tahir Ul Qamar M., Shokat Z., Muneer I., Ashfaq U.A., Javed H., Anwar F., Bari A., Zahid B., Saari N. (2020). Multiepitope-Based Subunit Vaccine Design and Evaluation against Respiratory Syncytial Virus Using Reverse Vaccinology Approach. Vaccines.

[50] Wiederstein M., Sippl M.J. (2007). ProSA-Web: Interactive Web Service for the Recognition of Errors in Three-Dimensional Structures of Proteins. Nucleic Acids Res..

[51] Pikkemaat M.G., Linssen A.B.M., Berendsen H.J.C., Janssen D.B. (2002). Molecular Dynamics Simulations as a Tool for Improving Protein Stability. Protein Eng. Des. Sel..

[52] Abraham M.J., Murtola T., Schulz R., Páll S., Smith J.C., Hess B., Lindah E. (2015). Gromacs: High Performance Molecular Simulations through Multi-Level Parallelism from Laptops to Supercomputers. SoftwareX.

[53] Burley S.K., Berman H.M., Kleywegt G.J., Markley J.L., Nakamura H., Velankar S. (2017). Protein Data Bank (PDB): The Single Global Macromolecular Structure Archive. Methods Mol. Biol..

[54] Grote A., Hiller K., Scheer M., Münch R., Nörtemann B., Hempel D.C., Jahn D. (2005). JCat: A Novel Tool to Adapt Codon Usage of a Target Gene to Its Potential Expression Host. Nucleic Acids Res..

[55] Cronan J.E. (2014). Escherichia Coli as an Experimental Organism. eLS.

[56] SnapGene | Software for Everyday Molecular Biology. https://www.snapgene.com/.

[57] Rapin N., Lund O., Bernaschi M., Castiglione F. (2010). Computational Immunology Meets Bioinformatics: The Use of Prediction Tools for Molecular Binding in the Simulation of the Immune System. PLoS ONE.

[58] Khalid K., Irum S., Ullah S.R., Andleeb S. (2022). In-Silico Vaccine Design Based on a Novel Vaccine Candidate Against Infections Caused by Acinetobacter Baumannii. Int. J. Pept. Res. Ther..

[59] ul Ain Q., Ahmad S., Azam S.S. (2018). Subtractive Proteomics and Immunoinformatics Revealed Novel B-Cell Derived T-Cell Epitopes against Yersinia Enterocolitica: An Etiological Agent of Yersiniosis. Microb. Pathog..

[60] Grandi G. (2010). Bacterial Surface Proteins and Vaccines. F1000 Biol. Rep..

[61] Barh D., Barve N., Gupta K., Chandra S., Jain N., Tiwari S., Leon-Sicairos N., Canizalez-Roman A., Rodrigues dos Santos A., Hassan S.S. (2013). Exoproteome and Secretome Derived Broad Spectrum Novel Drug and Vaccine Candidates in Vibrio Cholerae Targeted by Piper Betel Derived Compounds. PLoS ONE.

[62] Xu J., Zhang Y. (2010). How Significant Is a Protein Structure Similarity with TM-Score = 0.5?. Bioinformatics.

[63] De Vries S.J., Van Dijk M., Bonvin A.M.J.J. (2010). The HADDOCK Web Server for Data-Driven Biomolecular Docking. Nat. Protoc..

[64] Eisen R.J., Eisen L., Beard C.B. (2016). County-Scale Distribution of Ixodes Scapularis and Ixodes Pacificus (Acari: Ixodidae) in the Continental United States. J. Med. Entomol..

[65] Lyme BorreLiosis in Europe. https://caudwelllymedotnet.files.wordpress.com/2016/01/who-factsheet-lyme-borreliosis-epidemiology.pdf.

[66] Schoen R.T. (2020). Challenges in the Diagnosis and Treatment of Lyme Disease. Curr. Rheumatol. Rep..

[67] Sigal L.H., Curran A.S. (1991). LYME DISEASE: A MULTIFOCAL WORLDWIDE EPIDEMIC. Annu. Rev. Public Health.

[68] Stricker R.B., Johnson L. (2011). Lyme Disease: The next Decade. Infect. Drug Resist..

[69] Berende A., Ter Hofstede H.J.M., Vos F.J., Vogelaar M.L., Van Middendorp H., Evers A.W.M., Kessels R.P.C., Kullberg B.J. (2019). Effect of Prolonged Antibiotic Treatment on Cognition in Patients with Lyme Borreliosis. Neurology.

[70] Beaujean D., Crutzen R., Kengen C., Van Steenbergen J., Ruwaard D. (2016). Increase in Ticks and Lyme Borreliosis, Yet Research into Its Prevention on the Wane. Vector-Borne Zoonotic Dis..

[71] Batool M., Caoili S.E.C., Dangott L.J., Gerasimov E., Ionov Y., Piontkivska H., Zelikovsky A., Waghela S.D., Rogovskyy A.S. (2018). Identification of Surface Epitopes Associated with Protection against Highly Immune-Evasive VlsE-Expressing Lyme Disease Spirochetes. Infect. Immun..

[72] Khatoon N., Pandey R.K., Prajapati V.K. (2017). Exploring Leishmania Secretory Proteins to Design B and T Cell Multi-Epitope Subunit Vaccine Using Immunoinformatics Approach. Sci. Rep..

[73] Shey R.A., Ghogomu S.M., Esoh K.K., Nebangwa N.D., Shintouo C.M., Nongley N.F., Asa B.F., Ngale F.N., Vanhamme L., Souopgui J. (2019). In-Silico Design of a Multi-Epitope Vaccine Candidate against Onchocerciasis and Related Filarial Diseases. Sci. Rep..

[74] Pizza M., Grandi G., Telford J.L., Rappuoli R. (2002). Reverse Vaccinology: A Genome-Based Approach to Vaccine Development. Chim. Oggi.

[75] Bacchetta R., Gregori S., Roncarolo M.G. (2005). CD4+ Regulatory T Cells: Mechanisms of Induction and Effector Function. Autoimmun. Rev..

[76] Shamriz S., Ofoghi H., Moazami N. (2016). Effect of Linker Length and Residues on the Structure and Stability of a Fusion Protein with Malaria Vaccine Application. Comput. Biol. Med..

[77] Meza B., Ascencio F., Sierra-Beltrán A.P., Torres J., Angulo C. (2017). A Novel Design of a Multi-Antigenic, Multistage and Multi-Epitope Vaccine against Helicobacter Pylori: An In Silico Approach. Infect. Genet. Evol..

[78] Arai R., Ueda H., Kitayama A., Kamiya N., Nagamune T. (2001). Design of the Linkers Which Effectively Separate Domains of a Bifunctional Fusion Protein. Protein Eng. Des. Sel..

